# Pretreatment visceral metastases in castration resistant metastatic prostate cancer: role in prediction versus actual site of disease progression

**DOI:** 10.1186/s40644-022-00469-z

**Published:** 2022-07-14

**Authors:** Kathleen Ruchalski, Hyun J. Kim, Michael Douek, Steven Raman, Maitraya Patel, Victor Sai, Antonio Gutierrez, Benjamin Levine, Cheryce Fischer, Martin Allen-Auerbach, Pawan Gupta, Heidi Coy, Bianca Villegas, Matthew Brown, Jonathan Goldin

**Affiliations:** 1 Department of Radiological Sciences, UCLA, Los Angeles, CA USA; 2 UCLA Center for Computer Vision and Imaging Biomarkers, Los Angeles, CA USA; 3 Ahmanson Translational Theranostics Division, Department of Molecular & Medical Pharmacology, University of California Los Angeles, Los Angeles, CA USA

**Keywords:** RECIST, Disease progression, Prostate cancer, Visceral metastases

## Abstract

**Background:**

To evaluate the anatomic site(s) of initial disease progression in patients with castration resistant metastatic prostate cancer (mCRPC) in the presence or absence of pre-treatment visceral metastases while on systemic therapy.

**Methods:**

This is a retrospective cohort study of mCRPC patients who have baseline and at least one follow up bone scan and CT chest, abdomen and pelvis (CAP). Disease progression was determined by RECIST and/or ≥ 30% increase in automated bone scan lesion area score. Kaplan–Meier plot was used to estimate the median progression free survival and log-rank tests were used to compare anatomic sites.

**Results:**

Of 203 patients, 61 (30%) had pre-treatment visceral metastases. Patients with baseline visceral disease were 1.5 times more likely to develop disease progression (HR = 1.53; 95% CI, 1.03–2.26). Disease progression was a result of worsening bone scan disease (42% (16/38)) versus visceral (32% (12/38)) or lymph node disease (3% (1/38)) by CT or a combination thereof (23% (9/38)). Median time to progression (TTP) did not differ by anatomic location of initial progression (*p* = 0.86). Development of new lesions occurred in 50% of those visceral patients with soft tissue only progression and was associated with a significantly longer TTP (3.1 months (2.8–4.3 months) than those with worsening of pre-existing lesions (1.8 months (1.6–2.7 months); *p* = 0.04.

**Conclusions:**

Patients with pre-treatment visceral metastases in mCRPC are more likely to experience disease progression of bone disease with the initial anatomic site of progression similar to those without baseline visceral involvement.

## Background

Prostate cancer is the most common cancer diagnosed in men. In the setting of metastatic disease, androgen deprivation therapy is the initial treatment, however nearly all men eventually develop progressive disease; termed castration-resistant prostate cancer (mCRPC) [1, 2]. Clinical imaging, including radionuclide bone scan, computed tomography (CT) and magnetic resonance imaging (MRI) are used to assess treatment response in mCRPC in clinical care and trials [3–5]. The combination of these imaging modalities are usually used side-by-side to assess prostate cancer metastases, including bone and soft tissue disease (lymph node, local recurrence and visceral lesions).

The majority, 80–90% of patients with mCRPC develop osseous metastases, while approximately 36% develop lymph node metastases and 20–30% visceral involvement [6–9]. Visceral metastases are defined as soft tissue lesions involving liver, lungs, adrenal glands, peritoneum, pleura, brain and dura [10]. Visceral metastases occur less commonly than bone disease and the significance of their presence is less well studied [11]. However the clinical implications of these visceral lesions should not be underestimated. Visceral metastases tend to occur later in the disease course, and patients with visceral involvement have a worse prognosis than those with bone-only disease [6, 9, 11, 12]. Prior studies have shown that liver involvement portends the shortest overall survival when compared to lung, lymph node and/or osseous involvement [6, 13, 14].

There are increasing numbers of treatment options for mCRPC with growing numbers of post docetaxel options [11, 15]. Even patients with visceral involvement may derive a clinical benefit to systemic therapy with improvements in progression free and overall survival with treatment [8, 6, 14]. However, the actual anatomic site of radiographic disease progression when visceral metastases are present is not well understood. It is unclear if worsening of these visceral lesions is the sole driver of time to progression or if other soft tissue and osseous metastases equally contribute to significantly increasing tumor burden. The purpose of this study is to evaluate the anatomic site(s) of initial disease progression when visceral metastases are present prior to treatment in mCRPC patients.

## Materials and methods

### Patients and study design

Written informed consent was waived in this institutional review board-approved; Health Insurance Portability and Accountability Act-compliant, retrospective analysis. Patients who met the inclusion criteria for this study were identified from an anonymized imaging clinical trial database of patients with mCRPC in which imaging was serially obtained as part of clinical trials evaluating cabozantinib a tyrosine receptor blocker for outcome assessment between 2009–2018. Patients were excluded if (a) there was no CT and/or MRI Chest Abdomen and Pelvis (CAP) imaging or Technetium (Tc-99) bone scan at baseline, (b) no follow up imaging. Patients were classified as having visceral or no visceral disease. Additional sites of soft tissue involvement were further divided into the following anatomic sites: lymph node, local residual/recurrent disease, and soft tissue bone.

### Imaging acquisition

CT or MRI CAP and Tc-99 bone scan were performed at baseline and then every 6 weeks until radiographic progression or initiation of a subsequent anticancer therapy. Baseline and follow-up standard-of-care volumetric CT or MRI CAP studies were performed and reconstructed every 3–5 mm contiguously. Pre-contrast abdomen imaging as well as post contrast chest, abdomen and pelvis were performed unless intravenous contrast was contraindicated. A single phase (equilibrium phase) or dual phase (portal and equilibrium phase) of the abdomen and pelvis were obtained.

MRI studies were performed using a body coil with reconstruction every 3–5 mm without gap in the axial plane. Tc-99 whole body PA and AP bone scans were also acquired with 14–30 mCi (± 10%) of Methylene diphosphonate (MDP) and 2–4 h (± 30 min) acquisition post injection.

### Tumor assessment

#### Soft tissue disease

Soft tissue tumor response was assessed at all time points per RECIST 1.0 or RECIST 1.1 prospectively with blinded independent centralized review by two readers with adjudication by another reviewer in cases of disagreement [16]. The independent review committee consisted of readers in full time academic practice with collective experiences in prostate cancer and were blinded to all patient and clinical information. The presence of soft tissue disease was defined as measurable or nonmeasurable lesions per RECIST, with measurable disease including ≥ 10 mm long axis for organ based disease and ≥ 15 mm short axis for lymph nodes. A sum of diameters for target lesions was calculated and reported at baseline. All other anatomic sites of soft tissue disease were identified as non-target at baseline, and were categorized as lymph node, local recurrence/residual disease, visceral disease or soft tissue bone [17, 18]. Per RECIST, progressive disease was defined by the sum of diameters of target lesions with relative increase of at least 20% and with an absolute increase in size by at least 5 mm. Development of one or more new lesions was also considered progressive disease. Non-target disease was qualitatively assessed for unequivocal worsening of total non-target tumor burden [17, 18].

### Bone scans

Whole body Tc-99 PA and AP bone scans were analyzed using an algorithm that detects and measures the Bone Scan lesion area (BSLA) [19]. Two readers independently reviewed the BSLA segmentation output in a locked sequential reading paradigm. Each lesion identified by computer-aided detection could be accepted, modified, or removed. Additional lesions could be added by the reader if needed. All readers were blinded to patients’ clinical and biochemical status [20].

Bone disease progression was defined as a 30% or more increase in BSLA score or two or more new lesions in new locations when compared to baseline. Bone scan follow up was performed at 12 weeks to exclude any potential flare phenomenon [21].

### Statistical methods

Summary statistics for patient and lesion baseline characteristics were reported by visceral involvement groups. Wilcoxon rank sum tests were used to compare the difference between the baseline characteristics in those patients with visceral involvement and no visceral involvement. Frequencies and percentages were reported for baseline anatomic site of disease at the patient and lesion level by visceral involvement. Chi-squared test was used to compare the association in subjects with anatomic site of disease progression and visceral involvement. Kaplan–Meier plot was used to estimate the median time to progression in the anatomic sites of disease progression and log-rank tests were used to compare anatomic sites. Cox proportional models were used to estimate hazard ratio and 95% confidence interval. P values of less than 0.05 were regarded as statistically significant. All statistical analysis was performed using Stata (version 14.2; StatCorp, College Station, Tx).

## Results

### Patients and imaging

**Fig. 1:**
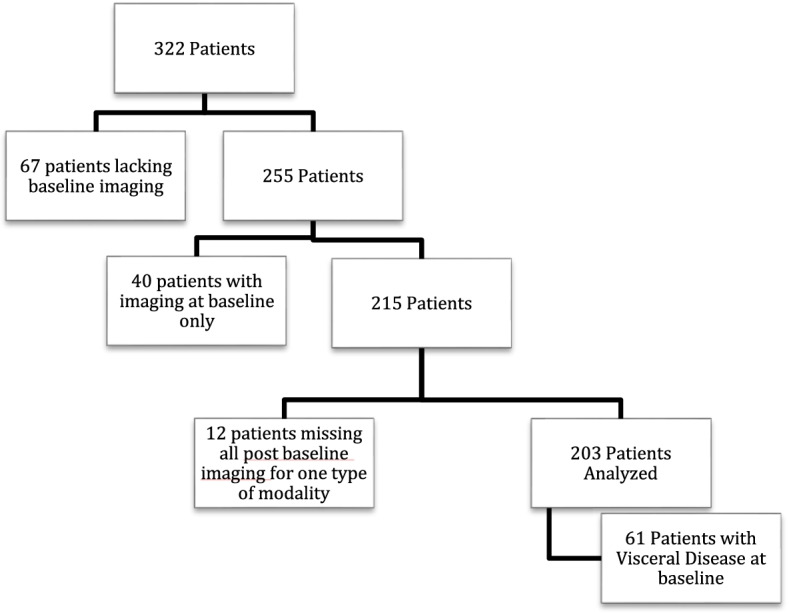
Flowchart for selection of the study population

Of the original 322 patients, 67 patients were excluded due to lack of baseline imaging and 52 patients were excluded due to lack of imaging after baseline studies. (Fig. 1). Therefore, for this exploratory analysis 203 patients with a mean age of 70 years (standard deviation (SD) 8.5 ± 10.0) were included. From this total cohort, 61 men had visceral metastases present on pre treatment baseline CAP imaging, and 142 had no visceral lesions present (Table 1). The mean number of follow-up visits was 2.6 CT CAPs (SD ± 1.9) and 2.6 bone scans (SD ± 2.0) for all 203 patients and was not significantly different when comparing those with or without visceral disease (*p* = 0.18 for CT CAPs and *p* = 0.22 for bone scan).

**Table 1:** Patient and lesion baseline characteristics

Characteristic	Total	Visceral involvement	No Visceral involvement	*p*-value
**(*n* = 203)**	**203**	**61**	**142**	
**Number of follow-up CT CAP **^a^	2.6 (1.9)	2.3 (1.9)	2.7 (1.9)	0.1786
**Number of follow-up BSLA **^a^	2.6 (2.0)	2.3 (1.9)	2.7 (2.0)	0.2193
Time to progression (months) ^b^	4.4 (3.0–8.5)	3.2 (2.7–6.8)	4.5 (3.2–8.8)	0.0329
**Number of patients with target lesion(s)**	158	61	97	
Average no. target lesion(s) on baseline ^a^	1.3 (1.5)	2.0 (1.6)	0.8 (1.3)	< 0.0001
**Average no. non-target lesion(s) on baseline **^a^	1.4 (1.0)	1.5 (1.2)	1.3 (0.8)	0.6527
BSLA score (mm2)^a^	88,916 (77,103)	86,989 (80,005)	89,958 (76,088)	0.6786

^a^ Data is the mean with standard deviation in parentheses

^b^Data is the median with interquartile in parentheses

### Imaging of metastases at baseline

#### Bone scan disease

Baseline total osseous tumor burden by bone scan was not statistically different amongst those with and those without visceral disease; with BSLA score of 86,989 mm2 in visceral and 89,958 mm2 in non-visceral patients (*p* = 0.68).

### Soft tissue disease

On average, patients with visceral involvement had 2 soft tissue lesions identified as measurable disease at baseline CT. Additional anatomic sites of disease involvement which did not meet measurable criteria were denoted as non-target lesions. Number of non-target lesions was similar amongst those with and without visceral involvement (1.5 non-target sites versus 1.3 non-target sites).

In those 61 patients  with baseline soft tissue disease, there were 126 target lesions and/or non-target lesions (Table 2). Of these, 59% (73/126) were visceral metastases; with the most common in the liver (26% (33/126)), lung (18% (22/126)), and adrenal glands (12% (12/126)). In addition to sites of visceral disease, bone lesions with soft tissue components, lymph nodes, and local residual/recurrent disease were also present in 14% (17/126), 27% (34/126), and 2% (2/126) of these subjects. In those subjects without baseline visceral disease, soft tissue lesions were identified in bone 47% (56/120), lymph nodes 50% (60/120) and as local residual/recurrent disease 3% (4/120).

**Table 2:** Anatomic sites of soft tissue metastases: Lesion level evaluation

Anatomic site of disease	Visceral involvement (*n*, %)	No Visceral involvement (*n*, %)
Total	126	120
Bone soft tissue	17 (13.5)	56 (46.7)
Local residual/recurrent disease	2 (1.6)	4 (3.3)
Lymph Node	34 (27.0)	60 (50.0)
Visceral disease	73 (57.9)	0
-Liver	33 (26.2)	0
-Lung	22 (17.5)	0
-Adrenal	12 (9.5)	0
-Spleen	2 (1.6)	0
-Peritoneal nodule	1 (0.8)	0
-Pleura	1 (0.8)	0
-Kidney	2 (1.6)	0

Nearly all patients with visceral disease also had concomitant bone metastases (Table 3). This included 44% (27/61) patients with bone and visceral disease, 51% (31/203) bone, visceral and lymph node disease, and 3% (2/203) with bone, lymph node, visceral disease and local recurrence. One patient with baseline visceral involvement lacked concurrent osseous disease but did have nodal metastasis.

**Table 3:** Baseline imaging characteristics: anatomic distribution of disease by patient

**Anatomic sites of metastases**	**Visceral involvement** **(*n*, %)**	**No Visceral involvement** **(*n*, %)**
**Total patients**	61 (30.1)	142 (70.0)
Bone only (BSLA and/or soft tissue bone)	0	80 (39.4)
**Bone and lymph node**	0	57 (28.1)
**Bone and local recurrence**	0	1 (0.5)
**Bone, lymph node and local recurrence**	0	3 (1.5)
**Bone and visceral disease**	27 (13.3)	0
**Bone, lymph node and visceral**	31 (15.3)	0
**Bone, lymph node, local recurrence and visceral disease**	2 (1.0)	0
**Visceral disease and lymph node**	1 (0.5)	0

In those patients without baseline visceral involvement, 56% (80/142) had osseous involvement only. An additional 40% (57/142) patients had a combination of bone and lymph node involvement, while 2% (3/142) had bone, lymph node and local recurrence and 0.7% (1/142) had bone and local recurrence.

### Evaluation of progression events

**Fig. 2:**
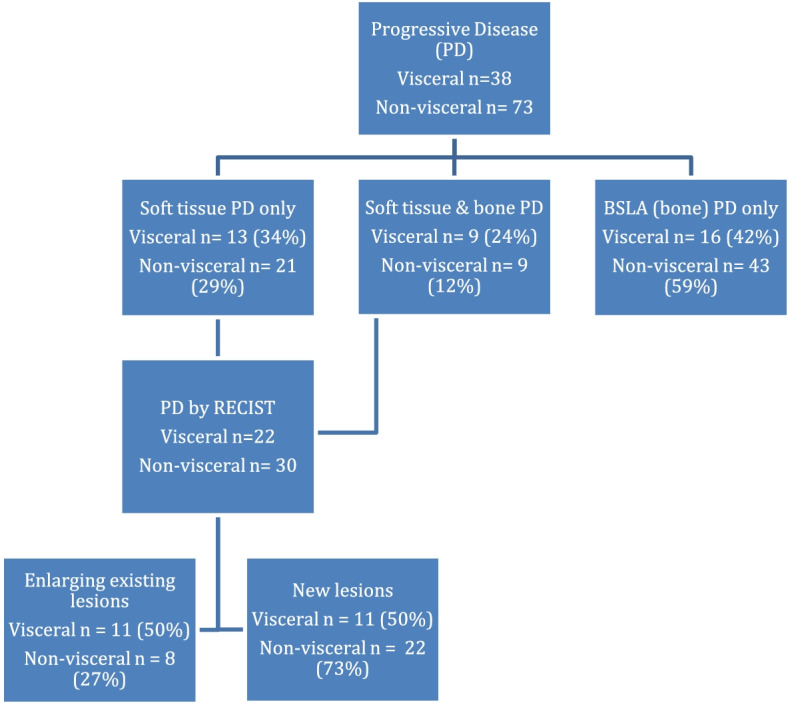
Flow chart of anatomic sites of disease progression for patients with or without baseline visceral metastases

Patients with baseline visceral disease had a significantly shorter time to progression than those without (3.2 versus 4.5 months, *p* = 0.04) (Fig. 1). Initial disease progression was due solely to worsening bone disease by BSLA in 42% (16/38) of patients (Table 4, Fig. 2). For 22 patients, disease progression was due to worsening soft tissue disease only and as a result of progressive lymph nodes only 3% (1/38) or visceral disease 32% (12/38). For 24% (9/38) of patients, progressive disease occurred simultaneously in both, bone scan disease and soft tissue locations.

**Table 4:** Initial source for disease progression: By anatomic site

**Anatomic site of disease progression**	**Number of patients** **(*n* (%))**		
	Visceral involvement	No visceral involvement	*p*-value
Bone (BSLA only)	16 (42.1)	43 (58.9)	0.006
Visceral disease	12 (31.6)	9 (12.3)	
Lymph node	1 (2.6)	12 (16.4)	
Multiple sites^*^	9 (23.7)	9 (12.3)	

^*^ Multiple sites: initial disease progression by RECIST which involves more than one type of anatomic site or simultaneous progression by RECIST and BSLA

In patients without baseline visceral disease, worsening of bone scan disease was also the most common cause of disease progression, occurring in 59% (43/73) of patients. Disease progression as a result of new or enlarging lymph node(s) occurred in 16% (12/73) patients, while new visceral disease was noted in 12% (9/73) patients.

**Fig. 3:**
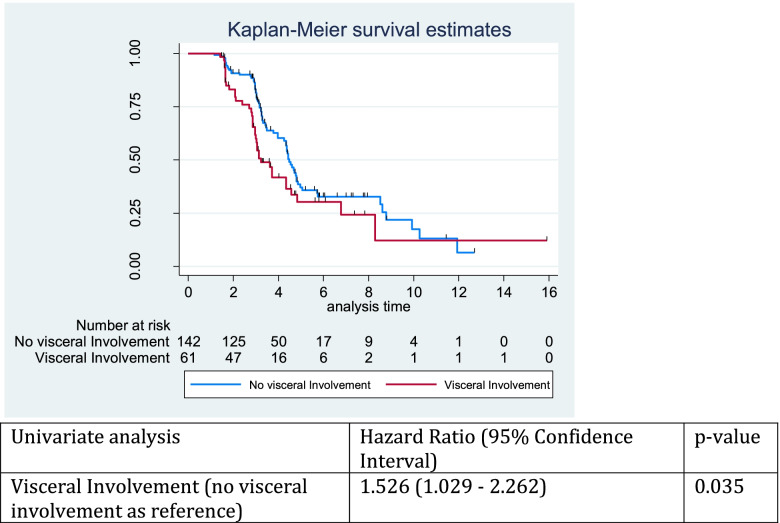
Time to progression in those patients with and without baseline visceral involvement

Patients with pre-treatment visceral disease were 1.5 times more likely to develop disease progression than those without baseline visceral involvement (HR = 1.53; 95% CI, 1.029–2.262) (Fig. 3). However time to progression was not statistically different when stratifying by initial anatomic site of disease progression, including bone, visceral, lymph node or multiple sites in either those patients with (*p* = 0.86) and without (*p* = 0.40) baseline visceral disease (Table 5).

**Table 5:** Initial source for disease progression: time to progression

	**Anatomic site of disease progression**				
**TTP** (months) **	**Bone (BSLA only)**	**Visceral disease**	**Lymph node**	Multiple sites^*^	***p*-value**
**Visceral involvement**	3.0 (2.1–3.6)	2.8 (1.6–3.1)	3.1	2.1 (1.6–3.2)	0.8639
**No visceral involvement**	3.3 (2.9–4.4)	4.2 (3.3–4.9)	3.0 (1.6–4.4)	3.2 (3.0–4.4)	0.3968

^*^ Multiple sites: initial disease progression by RECIST which involves more than one type of anatomic site or simultaneous progression by RECIST and BSLA

^a^Data is the median with interquartile in parentheses

*TTP* Time to progression

Soft tissue only disease progression occurred in 22 patients with baseline visceral lesions and 30 patients without pretreatment visceral metastases (Table 6). New lesions occurred in 50% (11/22) and 73% (22/30) of these patients with and without visceral involvement, respectively. Of those patients with pre-treatment visceral disease, progression due to presence of new lesions was associated with a significantly longer time to progression (3.1 months (2.8–4.3 months) than those with worsening of pre-existing lesions (1.8 months (1.6–2.7 months; *p* = 0.044).

**Table 6:** Initial source for RECIST only disease progression

**Lesion type for PD**	**Visceral involvement**		**No visceral involvement**	
	Number of patients (*n* (%))	TTP (months)	Number of patients (*n* (%))	TTP (months)
Enlarging existing lesion(s)	11 (50.0)	1.8 (1.6–2.7)	8 (26.7)	3.0(1.6–4.5)
Presence of new lesions(s)	11 (50.0)	3.1 (2.8–4.3)	22 (73.3)	3.8(3.0–4.8)
-multiple sites*	1 (4.5)	4.3	7 (23.3)	3.2(3.0–4.4)
-lymph node	1 (4.5)	3.1	6 (20.0)	1.9(1.8–4.4)
-visceral	9 (40.9)	3.0 (2.8–3.1)	9 (30.0)	4.2(3.3–4.9)

^*^Multiple sites: initial disease progression by RECIST which involves more than one type of anatomic site or simultaneous progression by RECIST and BSLA

*TTP* Time to progression

## Discussion

While there have been multiple prior studies characterizing the predictive and prognostic role of mCRPC visceral metastases, this is the first study to our knowledge that investigates how the anatomic sites of this disease burden individually contribute to radiographic disease progression overall.

**Fig. 4:**
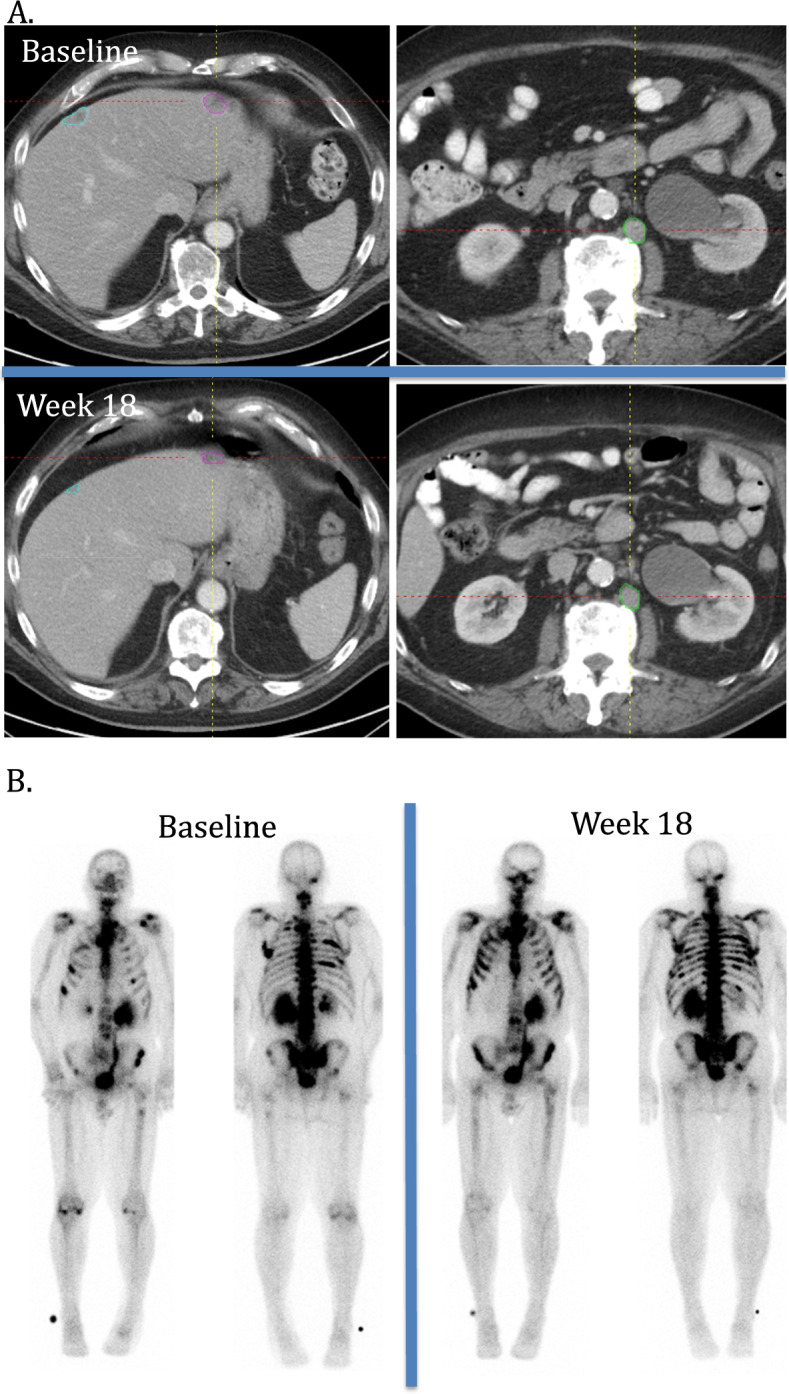
Example case: Baseline visceral disease with worsening disease by BSLA bone scan only as source of disease progression. **A**. Axial CT of the abdomen at baseline and week 18. Soft tissue target lesions at baseline include liver (blue and pink circles) and retroperitoneal lymph node (green circle) and were stable and slightly smaller at week 18. **B**. AP and PA Tc-99 bone scans were also performed at baseline and week 18. Multiple bone lesions are present at baseline, with increased and new lesions involving ribs bilaterally and right pelvis at week 18

Metastatic prostate cancer is known to have high inter- and intra- patient tumor heterogeneity with respect to site, that may impact treatment outcome [22–24]. There is both heterogeneity at baseline as well as during follow up with respect to site of progressive disease and new lesion development. Therefore, multimodality imaging to separately assess soft tissue disease and bone disease is usually combined to provide a single response to therapy (Fig. 4) [3, 24]. In our study the presence of visceral disease at baseline was associated with an increased risk of disease progression by either RECIST or bone scan assessment and a shorter time to progression. As a cause of disease progression, soft tissue progression was seen in both those with and without visceral involvement at baseline. New lesions occurred in 50% (11/22) and 73% (22/30) of these patients with and without visceral involvement, respectively. Of those patients with pre-treatment visceral disease, progression due to presence of new lesions was associated with a significantly longer time to progression (3.1 months (2.8–4.3 months) than those with worsening of pre-existing lesions (1.8 months (1.6–2.7 months; *p* = 0.044).

In our cohort, the presence of visceral disease was the main differentiating baseline imaging factor given that baseline bone scan disease burden was the same in those with and without pre-treatment visceral metastases. Even so, in patients with pre-treatment visceral disease, disease progression solely by worsening bone involvement occurred most often. While visceral lesions portend a poorer prognosis, they do not necessarily represent the site of worsening disease at the time of disease progression. Future work is required to further evaluate if visceral metastases themselves are predictors of poorer prognosis or are only a surrogate marker of larger baseline tumor burden. Larger pretreatment tumor burdens have also been previously shown in prostate cancer and other tumor types to portend poorer survival [25–27].

We note the following limitations. First, our analysis was limited to a retrospective analysis of prospectively interpreted images obtained from an anonymized imaging database. Second, during the original image review the independent review committee was blinded to clinical information during image interpretation, which may have limited how the qualitative component of non-target lesion progression was interpreted. Third, this study included only bone scan and CT/MRI chest, abdomen and pelvis. It should be noted that advances in next generation imaging, including whole body MRI and PET/CT with tracers targeting PSMA (PSMA PET/CT) may lead to new insights into the role visceral and bone metastases play in disease progression [28]. PSMA PET/CT can identify soft tissue disease not seen with anatomical imaging (e.g. metastatic nodes that are not pathologically enlarged) or bone scanning (bone metastases that have not elicited a sclerotic response), thus improving accuracy in prostate cancer staging and detection of metastases [29–31]. Similarly whole body MRI can provide both, anatomical and functional information in the setting of bone metastases, thus overcoming risk of flare phenomenon [23]. However these modalities are not yet commonly implemented into prospective drug therapeutic trials to be included in a similar analysis with respect to role of visceral metastases. Future use of PSMA PET/CT in therapeutic clinical trials and improved metastasis detection could lead to alterations in the prevalence of visceral metastases and better assess their role in disease progression.

## Conclusions

With ongoing advancements in therapies available for mCRPC, the development of mCRPC visceral metastases during a patient’s cancer treatment could theoretically continue to become more commonplace [11]. In this study, patients with pre-treatment visceral metastases were more likely to experience disease progression of bone disease, with this initial anatomic site of progression similar to those without baseline visceral involvement. Continued improved understanding of the role of mCRPC visceral lesions in patient outcomes will assist in treatment decisions for those patients with visceral metastases.

## Data Availability

This data is currently in a research database which is not publically available.

## Abbreviations

BSLA: Bone scan lesion area
CAP: Chest, abdomen and pelvis
CT: Computed tomography
mCRPC: Metastatic castration resistant prostate cancer
MDP: Methylene diphosphonate
MRI: Magnetic resonance imaging
PET: Positron emission tomography
PSMA: Prostate-specific membrane antigen
RECIST: Response evaluation criteria in solid tumors
Tc-99: Technetium
TTP: Time to progression
